# Chicago Veterinary College

**Published:** 1895-05

**Authors:** 


### COMMENCEMENT EXERCISES.

### CHICAGO VETERINARY COLLEGE.

On Thursday, March 21st, a banquet was given at the Sherman House by the Faculty of the Chicago Veterinary College to the graduating class, which proved a most enjoyable affair. Prof. Joseph Hughes acted as presiding officer on the occasion, and speeches were made showing the good-will existing between class and faculty. To the toast, "The Class of '95," Dr. George A. Lytle responded. "The Young Veterinarian in Practice" was done ample justice to by Dr. A. E. Flowers. "The College Association" was responded to by Drs. J. M. Lambert and A. McBride; "The Faculty," by Prof. A. H. Baker; and "Success in Life," by Major W. A. McCluer. Speeches were also made by Profs. George S. Baker, J. F. Ryan, E. L. Quitman, and C. E. Sayre. Letters were received from Profs. S. J. J. Harger, W. L. Zuill, E. A. A. Grange, D. A. Salmon, A. Liautard, T. D. Hinebauch, W. Horace Hoskins, Mayor Hopkins, Health Commissioner Reynolds, and John G. Shortall, President of the Illinois Humane Society, regretting their inability to attend.

The twelfth annual commencement exercises of the Chicago Veterinary College were held Friday, March 22d, and forty-two graduates received the diploma of the institution. The exercises were presided over by Prof. Joseph Hughes, who presented the Secretary's report for the past year. He stated that of the forty-two students who had just graduated, eighteen graduated with honors, of which the following is the list: G. F. McGuire, G. H. Chandler, W. A. Savage, R. Kammerer, A. E. Flowers, G. A. Lytle, A. McBride, J. Lambert, E. N. Nettleton, W. C. Baldwin, H. E. Talbot, B. H. Wood, H. O. Sanford, A. J. Payne, S. K. Hazlett, V. P. Smith, W. E. Bradley, E. G. Piper.

Dr. G. F. McGuire deserves special mention as having the highest general average. Dr. W. A. Savage received the prizes in Anatomy and Chemistry, and Dr. G. A. Lytle the prize in Theory and Practice. Having concluded his report the degree, "Doctor of Comparative Medicine" (M.D.C.), was conferred by the chairman on the members of the graduating class. Following the conferring of the degrees came the distribution of diplomas, and afterward the valedictory by Dr. C. I. Fleming. On the conclusion of the valedictory address Dr. J. J. McGrath, the class prophet, read an effusion which caused considerable merriment among his fellow-graduates. The doctorate address on behalf of the faculty, by Dr. A. H. Baker, completed the proceeding.

The following is a list of the graduates and their locations: H. G. Bailey, Hinsdale, Ill.; W. C. Baldwin, Roscoe, Ill.; F. C. Binnewies, Walworth, Wis.; R. S. Blackburn, Quincy, Ill.; W. E. Bradley, Lee, Mass.; W. H. Broderick, Chicago, Ill.; W. H. Button, Los Angeles, Cal.; C. H. Chandler, Marseilles, Ohio; B. L. Clarke, Monticello, Wis.; F. A. Crandall, Jr., Buffalo, N. Y.; A. E. Duckworth, Osceola, Ia.; C. I. Fleming, Terre Haute, Ind.; A. E. Flowers, Lyndonville, N. Y.; J. W. Golden, Jr., Sleepy Eye, Minn.; F. E. Greene, Lake Geneva, Wis.; A. H. Hartwig, Watertown, Wis.; S. K. Hazlet, Elgin, Ia.; I. W. Horton, Providence, R. I.; G. D. Judson, Polo, Ill.; R. A. Kammerer, St. Louis, Mo.; C. P. Kime, Montpelier, Ohio; P. O. Koto, Forest City, Ia.; F. S. Lambert, Du Page, Ill.; J. M. Lambert, St. Peter, Minn.; H. Leutholt, Taylor, Pa.; G. A. Lytle, Palatine, Ill.; A. McBride, Chicago, Ill.; J. J. McGrath, Chicago, Ill.; G. F. McGuire, Waterbury, Conn.; E. N. Nettleton, Chicago, Ill.; A. J. Payne, Wabash, Ind.; J. T. Pfeifer, Franklin, Wis.; E. G. Piper, Palmyra, Wis.; J. R. Sanders, New Sharon, Ia.; H. O. Sanford, Tyndall, S. D.; W. A. Savage, Lockport, Ill.; S. Sieker, Herman, Wis.; V. P. Smith, Jamestown, Ohio; H. W. Stern, Beaumont, Tex.; H. E. Talbot, Des Moines, Ia.; C. F. Van Blankensteyn, Brooklyn, N. Y.; B. H. Wood, Streator, Ill.

